# Relationship between Land Use/Land-Use Change and Human Health in Australia: A Scoping Study

**DOI:** 10.3390/ijerph17238992

**Published:** 2020-12-02

**Authors:** Tamzyn M Davey, Linda A Selvey

**Affiliations:** School of Public Health, The University of Queensland, Brisbane 4006, Australia; t.davey@uq.edu.au

**Keywords:** land use, land use change, environmental degradation, health, Australia

## Abstract

We undertook a scoping study to map the relevant evidence, summarise the findings, and to help identify gaps in the knowledge base on the relationship between land use/land-use change and human health in Australia. Our systematic search of the scientific literature for relevant articles up to August 2020 identified 37 articles. All 37 articles meeting our inclusion criteria were published after 2003. Zoonotic or vector-borne disease constituted the most common health outcome type studied. Agriculture/grazing was the land use/land-use change type most frequently represented in the literature, followed by coal seam gas extraction and open cut coal mining. The relationship between land use/land use change and human health in Australia, is not conclusive from the existing evidence. This is because of (1) a lack of comprehensive coverage of the topic, (2) a lack of coverage of the geography, (3) a lack of coverage of study types, and (4) conflicting results in the research already undertaken. If we are to protect human health and the ecosystems which support life, more high-quality, specific, end-user driven research is needed to support land management decisions in Australia. Until the health effects of further land use change are better known and understood, caution ought to be practiced in land management and land conversion.

## 1. Introduction

The planet’s natural systems are being transformed through human activity in ways that are profound, extraordinary, and accelerating [1,2,3,4]. Anthropogenic land use/land-use change (LULUC)—along with climate change and stratospheric ozone depletion—has, and continues to, degrade the environment to the extent that nearly every dimension of human health is implicated [1,2]. Given that the major causes of environmental degradation interact to impact human health, they are in effect, inextricable [1,2]. Informing responsible decision making that protects both human health and the health of the natural systems that support health, nonetheless requires an understanding of the effects of each of the major causes separately, in the relevant local context.

Like much of the globe, the majority of Australia’s landscape has been significantly altered (mostly in the last two and a half centuries) to accommodate population growth and growth economies through: urbanisation; agricultural/grazing expansion; forestry; mining; transport; waste management; and other industry, manufacturing, and infrastructure [5,6,7,8,9]. McFarlane et al. (2013) in an extensive examination of the association between infectious disease emergence and land-use and native vegetation change in Australia, reported that 22% of the infectious diseases they reviewed were associated with land conversion [10]. Furthermore, they found that the historical clustering observed in terms of incidence of environmental, vector-borne, and zoonotic disease followed periods of substantial land clearing in Australia [10]. This is supported by findings that suggest that land-use change increases the transmission of henipaviruses (like Hendra virus) in Australia [11,12]. Kessler et al. (2018) and Paez et al.’s (2018) reviews of the evidence on the topic of LULUC and spillover risk of henipaviruses to domestic animals and to humans, exemplifies the way in which LULUC can impact health. In the case of henipaviruses, the altered diet, roosting habitat, and migration behaviours of flying foxes, as a result of changes to their natural habitat, has increased the risk of disease for humans [11,12].

The evidence on the relationship between LULUC and human health in Australia has only relatively recently emerged—within the last two decades. The evidence is neither extensive or comprehensive in terms of coverage of LULUC and health topics, with a predominance of studies on the relationship between LULUC and vector-borne, or zoonotic disease. In order to adequately account for the risks to human health in decision making on land management and proposed land change, up-to-date summaries of the state of the evidence on the relationship between LULUC and health in Australia, are an essential starting point.

In this study, we aimed to assess the current state of evidence on the relationship between LULUC and human health in Australia, and to identify research gaps in the existing literature. We also aimed to draw implications from the existing evidence in order to inform policy and practice. To address this aim, we undertook a scoping study. Scoping studies are an appropriate approach where the ‘mapping’ of the relevant literature is the main goal [13]. Scoping studies typically address broad topics where different study designs and levels of evidence are applicable [13]. Unlike systematic reviews, scoping studies are less focussed on assessing the quality of the included studies [13]. Scoping studies can have a number of different purposes, but the main aim of this scoping study was to summarise the findings and range of research on the relationship between LULUC and human health in Australia. A summary of the evidence is a necessary starting point in terms of a synthesis of findings and to establish the state of the evidence, but also to help identify gaps in the knowledge base—all of which are necessary to ultimately inform practice and policy on land management and proposed land use change. Land management/proposed land use change decisions need to have at their core, a strong evidence base to support the protection not only of human health and wellbeing, but importantly, of the ecosystems that support life.

## 2. Methods

After Mastel et al. (2018) [14], we adopted for our scoping study the framework outlined by Arksey et al. (2005), [15] taking into account recommendations made by Levac et al. (2010): (1) identification of the relevant research question, (2) identification of relevant articles, (3) article selection, (4) charting of the data, and (5) collecting, summarizing and reporting of the results [13].

### 2.1. Identifying the Research Question

This scoping study answered the following question: What is the current state of evidence on the relationship between land use/land-use change and human health in Australia?

### 2.2. Identification of Relevant Articles

#### 2.2.1. Sources of Literature

We conducted a systematic search of the scientific literature for relevant articles in three electronic databases: two content specific databases—Medline/PubMed (biomedical sciences) and PsychINFO (psychology and related disciplines)—and one multidisciplinary database—Web of Science. We did not conduct a primary search for grey literature but included it where relevant, if it came to our notice in the reference lists of key articles.

#### 2.2.2. Search Terms

Apart from specifying place (Australia), the literature search included LULUC terms related broadly to different aspects and activities of land (and freshwater) use and change, and various types of human health outcomes/conditions/diseases. LULUC terms were derived from preliminary searches of the literature, and from key articles on the links between LULUC and health [2,4,14,16]. Similarly, health terms were drawn from key literature [2,4,10,17] and from Australian Health Department lists of nationally notifiable diseases [18]. Key search terms were mapped to each electronic database prior to the article search, with slight variations on some terms across the databases, depending on whether major terms were searched within the ‘subject heading’ or ‘thesaurus’ of a particular database. For example, “natural resources” is the broad subject heading in Medline/PubMed for the natural environment, while “nature (environment)” is the equivalent subject heading or “American Psychological Association Thesaurus” in PsychINFO. Table 1 shows the general terms used, not specific to each database. The search was carried out on March 19, 2019, at which time the citation and abstract for all articles identified via search terms were uploaded to an Endnote (Clarivate Analytics, Philadelphia, U.S.A) X9 library. The search was updated on 12 August 2020.

#### 2.2.3. Search Process

**Figure 1:**
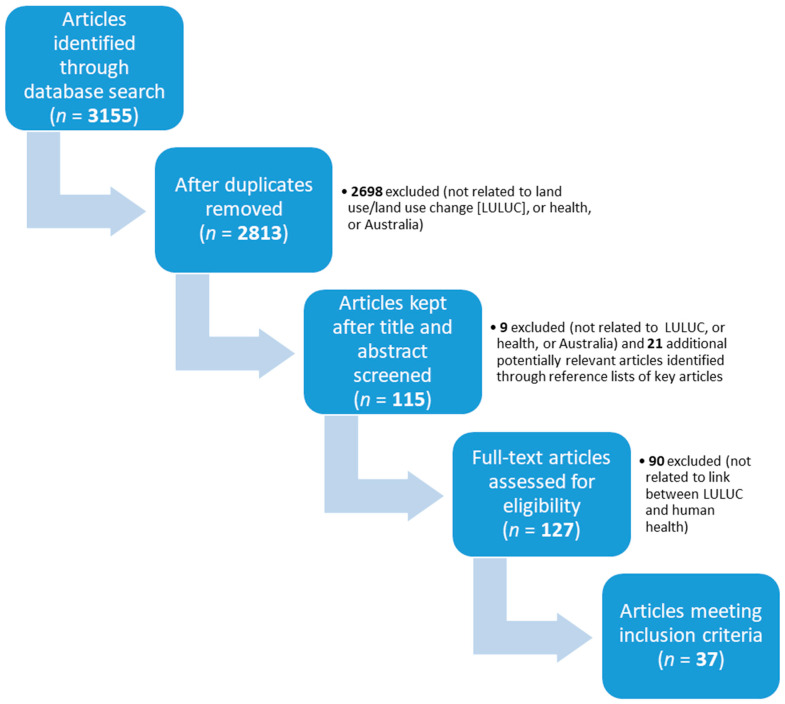
Flowchart of article search process (LULUC = land use/land-use change).

Our multi-step process for seeking relevant articles to answer the research question is outlined in Figure 1. Our initial search yielded 3155 articles. Two reviewers (the co-authors) were involved in deciding which articles based on title and abstract appeared sufficiently relevant enough to warrant full-text assessment for eligibility, and which articles finally met our inclusion criteria. At each step, the reviewers chose to eliminate or keep articles through a process of consensus. Thirty-seven articles ultimately met our inclusion/exclusion criteria.

### 2.3. Article Selection (Inclusion/Exclusion Criteria)

Publications meeting our criteria included: reports of original research (no reviews or opinion pieces) published up to 19 March 2019; in English; focused on or presenting data from Australia; that refer to a specific health issue; and include the topic of LULUC. The search was updated on 12 August 2020. No further eligible articles were identified.

Inclusion/exclusion criteria specific to the topic of LULUC was defined as either articles that related to one or more aspects of LULUC, or that partly or fully fulfilled the combined (land use, land cover, and land-use change) concept of LULUC as defined by the Intergovernmental Panel on Climate Change (IPCC). The ‘land use’ (LU) component was defined as, “the total of arrangements, activities, and inputs that people undertake in a certain land cover type”, with land cover as, “the observed physical and biological cover of the earth’s land, as vegetation or man-made features’ [19]. The ‘land-use change’ (LUC) component was defined as, “…human activities which: (a) change the way land is used (e.g., clearing of forests for agricultural use, including open burning of cleared biomass), or (b) affect the amount of biomass in existing biomass stocks (e.g., forests, village trees, woody savannas, etc.)” [19].

Inclusion/exclusion criteria specific to the topic of human health included articles that focused on either direct health outcomes as a result of LULUC, for example an infection or depression; or causative agents of disease/ill health, for example prevalence of mosquitos or pollution exposure, even where no health measurements were taken. While the presence or absence of causative agents does not always result in ill health, we were interested in capturing published literature on increased risk—even if inexact—of disease in humans in relation to LULUC.

We included studies looking at both positive as well as negative links between LULUC and human health. Notwithstanding the inextricable links between the different forms of environmental degradation, i.e., LULUC and climate change, we excluded studies focusing on climate change/weather extremes and health, because there already exists a relatively large body of evidence on this topic. Articles meeting content/topic criteria were then assessed on the basis of a combination of study objectives and whether the health and LULUC topics were sufficiently the focus of the study and adequately addressed in the results and discussion sections of the articles.

### 2.4. Data Management and Characterization/Charting

We charted data extracted from all articles meeting the inclusion criteria, in a Microsoft Excel (2016) spreadsheet. Included was: author/s; year of publication; title; research objectives; location; study design; type of LULUC and measures; health outcome and measures; and summary of findings. These data points were selected specifically to answer the research question and meet the study purpose.

## 3. Analysing, Summarizing, and Reporting the Results

Using the above described charted data as the basis of the analysis, we undertook to quantify the extent, nature and distribution of the studies included in the review (descriptive quantitative analysis). We also organised the literature thematically according to the different types of LULUC and their relationship to human health outcomes (qualitative analysis).

### Results

Profile of Included Literature

Table 2 summarises the extent, nature, and distribution of the 37 articles included in the analysis. Of the articles meeting our inclusion criteria, the earliest was published in 2004. There were four times the number of articles published in the 2016-2019 period (*n* = 12, 32.4%), compared with that of the 2004-2007 (*n* = 3, 8.1%) period (Table 2). The vast majority of articles described studies which had employed quantitative methods (*n* = 31, 83.8%) (Table 2).

All Australian states and territories, except South Australia and The Australian Capital Territory, were represented in the included articles, but there was unequal representation across and within states and territories. The State most frequently represented was Queensland, constituting 32.4% of all articles (*n* = 9 focussing just on Queensland, and *n* = 3 focussing on eastern Australia which includes Queensland and New South Wales). New South Wales (*n* = 8, 21.6%, including State-specific, Eastern, and Southeast Australia-located studies) and Western Australia (*n* = 6, 16.2%) were the next most frequently represented locations in Australia (Table 2).

Over one third (*n* = 13, 35.1%) of all articles focused on zoonotic or vector-borne disease (Hendra virus, Ross River virus, and mosquito-borne disease—general), with a majority of those specific to Hendra virus and Ross River virus, each constituting *n* = 4, 10.8% of all articles included (Table 2). The vast majority of zoonotic or vector-borne disease articles (*n* = 11, 84.6%) measured vector abundance or animal disease as a proxy for human disease, i.e., not as a direct measure of human disease (Table 2). Just less than one third of all articles related to health and wellbeing in general or multiple health outcomes (*n* = 12, 32.4%), with the remaining one third of articles dedicated to mental health, ecosystem goods and services, melioidosis, pathogen spillover—general, pollen counts, and respiratory disease (Table 2). Over half of all articles (*n* = 21, 56.7%) based their health outcome of interest on observational data, e.g., incidence, prevalence, mortality, counts of bacteria or pollen, or quantity/quality of the relevant ecosystem goods and services (soil, water, biodiversity) (Table 3). Self-report survey (*n* = 7, 18.9%), and semi-structured interview (individual and/or focus group) (*n* = 4, 10.8%) were the next most frequently employed methods for assessing the respective health outcomes of interest (Table 3).

The most frequent LULUC topic among the included articles was multiple land use/change types (*n* = 17, 45.9%); in these articles two or more land use/change types were compared in relation to the respective health outcome, and frequently but not always included single or multiple agricultural land use types (Table 2). Dryland salinity (*n* = 5, 13.5%), coal seam gas extraction (also *n* = 5, 13.5%), and open cut coal mine (*n* = 3, 8.1%) were the next most frequent LULUC topics (Table 2). Just less than half (*n*= 16, 43.2%) of all articles based their LULUC topic of interest on existing land use (GIS mapping and satellite images) or field data (observations, field-surveys) (Table 3). For 15 (40.5%) articles, the LULUC topic of interest did not require data or measurement as it related to either proposed mining or agriculture development or existing mining, contaminated sites, urbanisation, or traditional Indigenous ancestral lands (Table 3).

## 4. Summary of the Literature: Relationship between Land Use Change and Health in Australia

### 4.1. Agriculture/Grazing

Two articles focussed specifically on agricultural land use [21,41]. An additional seven articles did not focus on agricultural land use/change alone, but included agriculture/grazing as one of a number of land use types used for comparison in relation to the respective health outcome of interest. (Table 3) [25,28,33,35,39,50,55].

### 4.2. Dryland Salinity

Secondary salinity occurs over time as a result of agricultural activity in dry environments such as southwestern Western Australia, where the native vegetation has been cleared and replaced with shallow-rooted crops and grazing grasses [26]. Shallow-rooted vegetation draws less on groundwater, which can lead to a rise in the water table, transporting land and surface water-contaminating salts to the surface [26]. Although the phenomenon of secondary salinity necessarily relates to agricultural land use/change, the five articles which focused on various health outcomes related to dryland salinity—‘mental health’; mosquito abundance’; and ‘other chronic disease’—are presented in their own category because of the specific conditions (and health outcomes) of salinity as a LULUC type (Table 3).

### 4.3. Anthropogenic Land Change—General

Table 3 summarises findings of the eleven articles which measured ‘converted’ versus ‘unconverted’ land, [52] or multiple land use types, in relation to ‘mosquito abundance’; ‘pollen counts’; ‘respiratory disease’; ‘vector-borne and infectious disease’; and ‘wellbeing’ [29,34,42,43,45,47,49,51,53,54].

### 4.4. Urbanisation

One article focussed specifically on urbanisation as a LULUC type, and assessed the risk of Hendra virus outbreaks in Australia as a result of year-round alternative food for flying foxes in expanding urban-and peri-urban settings and a contraction of natural food sources (Table 3) [30].

### 4.5. Contaminated Sites

The only article addressing the impacts of contaminated sites (not related to contamination from mining operations) assessed wellbeing as an outcome (Table 3) [56].

### 4.6. Mining

Eight articles assessed the relationship between mining and health outcomes in Australia. Five were specifically related to coal seam gas (CSG) extraction, [36,38,44,46,48] with the remaining three pertaining to open-cut coal mining [21,32,37]. The five articles which focussed on CSG extraction, variously measured general health, [36,38,48] mental health, [46] or wellbeing [44] as an outcome (Table 3). All three open-cut coal mining articles measured wellbeing as the health outcome (Table 3) [21,32,37].

### 4.7. Traditionally Owned/Ancestral Indigenous Land Managed by Indigenous Communities

Two articles identified as part of our search process address the importance of access to ancestral lands for the general health and wellbeing of Australian Indigenous communities (Table 3) [22,27].

## 5. Discussion

Of the 37 articles meeting our inclusion criteria (not restricted by publication date), all were published after 2003. Most of the research focussed on the eastern states of Queensland and New South Wales, and on specific regions within the states and territories. Zoonotic or vector-borne disease constituted the most common health outcome type studied—well over a third of all articles. Agriculture/grazing was by far the LULUC type most frequently represented in the literature we reviewed, followed by coal seam gas extraction and open cut coal mining.

### 5.1. Land Use/Land-Use Change and Human Health in Australia

Agriculture/grazing was reported by the range of studies in the review as having negative, inconclusive, and neutral relationships with health outcomes in Australia. In the literature, agriculture/grazing was related to decreased wellbeing [39,40]; increased abundance of disease-transmitting mosquitoes [20,23,24,28]; decreased ESGs [36]; occurrence of the melioidosis bacterium *B. pseudomallei* in soil [25,33]; and hospitalisations for depression [26,31]. The relationship between agriculture/grazing and soil quality [51]; and agriculture/grazing and water quality, Ref. [55] was mixed/inconclusive. There was no association between mental health scores in women, and soil salination, in a study conducted in the rural south-western corner of Western Australia [41] The mixed and sometimes conflicting findings epitomise the complexity of the relationship between LULUC and human health, and the challenge of synthesising findings, largely because of the variety of methods used. For example, studies looking at the association between mental illness and dryland salinity in the same area in Western Australia came to different conclusions on the basis of different methods. Dryland salinity can have economic and social impacts on communities, with changes in agricultural productivity leading to financial stress and potentially mental illness, but Fearnley et al. (2014)—using self-reported survey data as part of an established longitudinal study of a randomly recruited study sample from the national Medicare health insurance database, which covers all citizens and permanent residents of Australia—found no associations between salinity and mental health scores for women across three age cohorts [41]. By contrast, Speldewinde et al. (2009) and Speldewinde et al. (2011) reported findings which indicated that after adjustment for major socioeconomic and demographic factors, an elevated risk of hospitalisations for depression was associated with residence in areas proportionately more affected by dryland salinity [26,31]. In explaining findings inconsistent with their own, Fearnley et al. (2014) proffered that area level measurements of aspects of the physical environment—such as salinity—may be poor indicators for individual health outcome analyses due to ‘ecological fallacy’, which is commonly observed in spatial analysis, i.e., the individuals being admitted to hospital may not be the same individuals exposed to dryland salinity [41]. Differences in results between Fearnley’s et al. (2014) and the two Speldewinde (2009, and 2011) studies on salinity and depression may also be due to the use of different measures for mental health: the Mental Health Component Score (MCS) versus the use of hospital admission data, respectively [41]. While one would expect some correlation, the MCS—a general measure of mental health— is unlikely to capture exactly the same phenomenon as hospital admissions data, [41] in terms of both severity and specificity of mental health conditions.

Other anthropogenic LULUC types such as industry, non-native grasslands, and urbanisation—all of which necessarily impact biodiversity—were negatively associated with health in terms of increased mosquito abundance and species richness [34,47,49,54]; Hendra virus spillover to horses and humans [30,45,51]; and respiratory disease [54]. However, as with agriculture/grazing, the evidence indicated a nuanced relationship between the ‘other anthropogenic LULUC types’ and human health. For example, the highest risk of pathogen spillover (not disease specific, in the case of Faust’s et al. (2018) simulation study) from wildlife to domestic animals, to humans, occurs at intermediate levels of habitat loss [52]. Interspecies contact and host populations not only vary with the proportion of land converted; time since initial habitat loss is also likely to drive changes in infectious disease transmission [52]. The working explanation for the dynamic relationship between land change and pathogen spillover—using mosquito-borne disease as an example—is that declining resources for reservoir hosts (wildlife) in converted landscapes drive wildlife from their natural habitat, leaving infected vectors (mosquitoes) to attain blood meals from the (now) more readily available human hosts [52]. Pathogen spillover is complex and influenced by multiple processes, including pathogen dynamics in reservoir hosts, environmental processes determining pathogen survival, carriage of the pathogen beyond hosts, and the behaviour and susceptibility of recipient hosts [10,11,12,57]. Each one of these processes may respond to changing landscapes and determine the particular relationship between land conversion and disease emergence [52].

Evidence for the relationship between mining—another anthropogenic LULUC type—and health in Australia also signified a complex dynamic, with competing vested interests, and differential impacts and benefits. Coal seam gas extraction was found to be associated with depression and stress in farmers [46]; increased population hospitalisation rates for neoplasms (tumours) and blood/immune disease [48]; and various health complaints—skin and eye irritations, headaches, paraesthesia, fatigue, and difficulty concentrating [36]. However, these findings were contentious, with as many studies on CSG extraction and open cut coal mining reporting inconclusive or mixed findings with regard to impacts on health in Australia [32,37,38,44]. For example, The McCarron (2013) investigation which reported various health complaints of residents in a CSG extraction area in Queensland, [36] was in response to a State health department report which concluded no clear link between residents’ health complaints and the local CSG activities [38] McCarron (2013) surveyed 113 residents from 38 households who had previously expressed concerns about health impacts of the CSG activity, and collated their self-reported health status [36]. As part of the study, neither clinical nor environmental sampling assessments were undertaken, and the sample was not representative of the whole population [36]. While the Queensland Health report indicated that no medical staff visited the CSG site as part of their investigations, and only 15 people were clinically examined, they reported that there was nothing in their environmental assessments on air, water, or soil to indicate a risk to human health [38]. McCarron’s (2013) conclusions do align more closely, than do the Queensland Health (2013) report, to a summary of literature from Australia and overseas regarding human and environmental health concerns from unconventional gas mining [58]. Notwithstanding incomparable methods, and methodological limitations in the two reviewed articles reporting conflicting findings, they serve to highlight the challenges in undertaking studies involving CSG exploration and health outcomes in Australia, including the small sizes of the affected populations and highly politically-charged situations.

Another complexity highlighted in the available evidence on the relationship between land use type, and human health in Australia, was the finding of ‘opportunity-threat’ differentials within affected groups. For example, while a proposed open cut-coal mine development in Queensland was a source of stress (‘threat’) to a range of community members—but especially to farmers and their families—some in the community saw ‘opportunity’, and this differential created tension between groups [37]. Similarly—albeit related to agricultural development—Adams et al. (2014) identified differences in responses by residents to the proposed development of a river catchment area in the Northern Territory. Indigenous respondents were more likely to value socio-cultural and biodiversity factors over the ‘commercial’ factors of the proposed development, while respondents who earned an income from agriculture were more likely to value the economic gains from which they were likely to benefit [40]. There was also evidence in the literature we reviewed, of differential impacts on human health and wellbeing over time in relation to land use change. This was epitomised by the outcomes of a choice modelling exercise with residents in the Hunter Valley Coalfields of New South Wales, in relation to the proposed extension of an open-cut coal mine. Community well-being declined with increased clearing of endangered ecological communities, loss [59] of highly significant Aboriginal sites, and displacement of rural families as a result of the mine’s extension, but increased with the length of time that the mine provided employment and with the planting or protection of endangered ecological communities as offsets [32].

Both articles on the relationship between traditionally-owned ancestral land and health showed a positive association [22,27]. Traditionally-owned ancestral lands are unlike other LULUC categories in this scoping study in that they are not focussed on a change from unconverted to converted landscapes/ecologies/ecosystems which may impact human health and wellbeing. Rather, they evaluate the relationship between *access* to this ‘land use type,’ and health. While all of Australia is technically ‘traditionally-owned’—ancestral land was never ceded to colonists when they settled in Australia in the late eighteenth century—only a proportion of the continent is currently recognised in common law as being traditionally-owned. Access to ancestral lands for Aboriginal and Torres Strait Islander peoples is either through a grant of freehold or perpetual lease title (land rights) or through native title which arises as a result of the “recognition of pre-existing Indigenous rights and interests according to traditional laws and customs” [60]. In the case of native title, Indigenous groups may have customary rights to areas with other land tenure, such as agriculture or National Park land [22]. By implication, conversion of traditionally-owned ancestral land, while it may not alter access, would have implications for the health of the traditional owners themselves, given the inextricable link between the health of the land and the health of Indigenous Australians [59,61,62].

### 5.2. Limitations of the Scoping Study Methods

We used a systematic and comprehensive search process, and adhered to an established scoping study framework. Notwithstanding this, the broad nature of the research question, and the fact that the research topic necessarily involves multiple scientific disciplines—often employing different terminology—meant that it was possible that some relevant articles were not captured in our search process. Within those limits, we nonetheless were able to fulfil the aims of our scoping study to summarise the range of current research reports and the relationship between LULUC and health in Australia, and identify broad gaps and implications for practice and policy.

### 5.3. Limitations of the Literature Reviewed

The evidence on the relationship between LULUC and human health in Australia is necessarily complex, with a multiplicity of land use types, health outcomes, and methods used. Some studies compared converted and unconverted land use types in relation to a health outcome, while others focussed on the relationship between one already converted land use-type/proposed land use change, in relation to a health outcome. Primarily because of the nature of the topic, there were no studies assessing causation, only association, which limits the strength of the conclusions that can be drawn from the findings. Human health states have multiple, interacting causes and comorbidities, and effects of a particular type of LULUC may only be relevant to that particular place, with findings unable to be generalised. The study of LULUC is also inherently complex, both in terms *how* it interacts with other factors—especially climate change—to impact health, and the *ways* in which it can impact health. Landscape changes can both exacerbate climate change, for example, deforestation which reduces carbon dioxide sinks can contribute to warming, and/or LULUC can be exacerbated by climate change (for example, loss of vegetation from drought) [3,4]. In combination, LULUC, climate change, and stratospheric ozone depletion can impact human health and wellbeing either directly (for example, through heatwaves or exposure to pollutants), indirectly (for example, through livelihood loss or population displacement), and/or through the mediation of compromised ecosystems and ecosystem ‘goods and services’ (EGSs), such as reduced food yields or altered infectious disease risk [2,17]. Unless climatic factors (which measured at a single point in time may not necessarily be representative of anthropogenic climate change) are measured alongside LULUC, it may be difficult to confidently attribute the measured health impacts to LULUC alone. Two of the studies we reviewed, did also measure climatic factors. Haberle et al. (2014) assessed the macroecology of airborne pollen in urban areas, Ref. [42] and reported that the only statistically significant factor explaining the difference between airborne pollen in each of the 11 sites studied was minimum temperature and mean annual precipitation [42]. Similarly, it was found that the critical ecosystem services functions associated with decomposition and nutrients cycling declined with increasing aridity (dryness/lack of precipitation), and that the effects of aridity were of a greater magnitude than any effects due to grazing [50].

### 5.4. Gaps and Implications for Research, Practice, and Policy

The current scoping study adopted a broad, all-LULUC-all-health outcomes-in-Australia approach, with the primary purpose of summarising and disseminating the findings and the full range of research. It also sought to identify research gaps; the fulfilment of which might ultimately inform policy and practice. In deliberations over a specific land management case—for example, land clearing/deforestation to facilitate agricultural development—practice and policy is likely to be best served by a synthesis of evidence of the impacts on health of that particular LULUC type. While there were 14 papers that described the health impacts of agriculture and grazing (including dryland salinity, a consequence of both), the studies were largely cross-sectional and therefore unable to ascribe causation. In spite of the large impact that agriculture has on water availability and water quality, this remains a large gap in the literature. For example, we found no studies examining the impact of loss of biodiversity, wetlands and rivers on health, in spite of these being major consequences of irrigation [63,64]. We also did not find any studies examining the health impacts of agriculture on microbiological water quality (for example, *Cryptosporidium parvum* contamination), [65] or any studies investigating the link between agriculture, land degradation and human dust exposure [66,67,68]. Similarly, more prospective studies in relation to the impact of mining are required, together with those that examine longer term impacts. Studies related to the health impacts of urban incursions into natural areas are also limited. General research gaps include geographical representation across and within all states and territories in Australia, and work on the health effects of LULUC which impacts food yield, quality of food, livelihood loss, and population displacement.

The work is specific to the Australian context, but the scoping study methods are necessarily relevant to all contexts. General findings from the current study may be reflective of the state of the evidence for other contexts and would depend on the nature of the research question; for example, whether a broad all LULUC-types and health outcomes approach was taken—as in the current study.

## 6. Conclusions

The relationship between land use/land use change and human health in Australia is not conclusive from the existing evidence. This is because the topic has not been investigated comprehensively across the whole continent. We also found conflicting results between some of the studies, perhaps reflecting the complexities of the relationships between LULUC and human health. More high-quality, specific, end-user driven research is needed to support land management decisions in Australia—in particular, to consider the human health and the ecological implications of land use changes. During a time of rapid deforestation in many parts of Australia, this work is critically important.

## Figures and Tables

**Table 1:** Key terms with synonyms and syntax used for literature search.

#1 Place Term	#2 Land Use/Land-Use Change Terms	#3 Human Health Terms	Combined Search
Australia	“land use change” OR “land cover change” OR “land clear*” OR “land use” OR “vegetation clear*” OR “land* change*” OR “land degrad*” OR “environment* OR degrad*” OR “eco* degrad*” OR “forest degrad*” OR “forest loss*” OR deforest* OR “environment* change” OR mine OR mining OR agriculture* OR farm OR road OR dam OR urban* OR “natur* environment*” OR “eco* services” OR eco* OR livestock OR irrigation OR desert*	disease OR health OR health status OR respiratory tract diseases OR allerg* OR immun* OR zoono* OR *virus* OR leptospirosis OR ornithosis OR dengue OR malaria OR encephalitis OR melioidosis OR ulcer OR *gattii OR “photorabdus asymbotica” OR *fever OR typhus OR *granulosus OR parasite OR anthrax OR brucellosis OR tularaemia OR mental OR health OR well-being OR “mental illness” OR “mental disorders” OR “nutritional status”	#1 AND #2 AND 3

**Table 2:** Profile of included literature.

Article Characteristics	Number (*n* = 37) of Included Articles, *n* (%)	Article Number in References
Publication year		
2004–2007	3 (8.1)	[20,21,22]
2008–2011	9 (24.3)	[23,24,25,26,27,28,29,30,31]
2012–2015	13 (35.1)	[32,33,34,35,36,37,38,39,40,41,42,43,44]
2016–2020	12 (32.4)	[45,46,47,48,49,50,51,52,53,54,55,56]
Study Type		
Mixed	1 (2.7)	[40]
Qualitative	5 (13.5)	[21,22,27,37,38]
Quantitative	31 (83.8)	[20,23,24,25,26,28,29,30,31,32,33,34,35,36,39,41,42,43,44,45,46,47,48,49,50,51,52,53,54,55,56]
Location in Australia		
Australia-wide	3 (8.1)	[46,53,54]
Australia-wide urban specific	2 (5.4)	[42,56]
Eastern Australia (area (multi-State))	3 (8.1)	[30,43,51]
New South Wales	3 (8.1)	[21,32,49]
Northern Territory	5 (13.5)	[22,25,33,39,40]
Queensland	9 (24.3)	[34,36,37,38,44,45,47,48,55]
Southeastern Australia (area (multi-State))	2 (5.4)	[29,50]
Tasmania	1 (2.7)	[28]
Victoria	2 (5.4)	[27,35]
Western Australian (State)	6 (16.2)	[20,23,24,26,31,41]
*No location* (model)	1 (2.7)	[52]
Health topic		
Ecosystem goods and services	3 (8.1)	[35,50,55]
Health and wellbeing—general (not disease specific) or multiple health conditions	12 (32.4)	[21,22,27,29,31,32,36,38,39,40,44,48]
Hendra virus	4 (10.8)	[30,43,45,51]
Melioidosis	2 (5.4)	[25,33]
Mental health-specific	5 (13.5)	[26,37,41,46,56]
Mosquito-borne disease—general	4 (10.8)	[20,34,47,49]
Pathogen spillover—general	1 (2.7)	[52]
Pollen counts	1 (2.7)	[42]
Respiratory disease	1 (2.7)	[53]
Ross River virus	4 (10.8)	[23,24,28,54]
LULUC topic		
Agriculture-specific	2 (5.4)	[20,40]
Anthropogenic land change—general	1 (2.7)	[52]
Coal seam gas extraction	5 (13.5)	[36,38,44,46,48]
Contaminated sites	1 (2.7)	[56]
Dryland salinity	5 (13.5)	[23,24,26,31,41]
Multiple land use/change types	17 (45.9)	[25,28,29,33,34,35,39,42,43,45,47,49,50,51,53,54,55]
Open cut coal mine	3 (8.1)	[21,32,37]
Traditionally owned Indigenous land	2 (5.4)	[22,27]
Urbanisation	1 (2.7)	[30]

**Table 3:** Summary of articles which met inclusion criteria for the Scoping Study.

Citation	Location	LULUC Type and Measure	Health Outcome and Measure	Findings Summary
**Agriculture/Grazing**				
Impact on Ecosystem Goods and Services, Including Water and Soil Health				
Baral et al. 2013 [35]	300 km^2^ sub-catchment of the Lower Glenelg Basin, southwestern Victoria, Australia	Multiple; based on Land Use and Management classification data, and vegetation classes	Various ecosystem goods and services (EGSs)—timber production, carbon stock, provision of water, water regulation, biodiversity, and forage production; via various existing data	Changes of pasture to managed plantation since the 1970s increased provision of most EGSs. However, compared with pre European condition of intact native vegetation (significantly reduced after conversion to pasture from 1850s) there has been significant loss in total provision of EGSs, coupled with increased demand.
Vandandorj et al. 2017 [50]	Southeastern Australia	Multiple; via a Geographical Information System	Soil health and plant production; via soil health index, observation, and field measures	Effects of grazing on ecosystem services were herbivore specific and varied. Critical functions associated with decomposition and nutrients cycling declined with increasing aridity, and these effects were of a greater magnitude than any effects due to grazing. Livestock grazing resulted in reductions in plant biomass, but was associated with greater nitrogen availability (sheep, and to a lesser extent cattle) and decomposition. There was an overall neutral effect of grazing on carbon storage. Phosphorus cycling was one of the ecosystem services less affected by grazing intensity.
Wijesiri et al. 2018 [55]	Ningi Creek catchment, North Brisbane Statistical Division, Queensland, Australia	Multiple; data from ‘Queensland Spatial Catalogue’	Water quality; via surface water samples	Majority of pollutants released into catchment waters came from ‘Water environment’ land use type (marsh, wetland, and other water bodies ecosystems). Concentrations of iron and nitrates in downstream water environment may have been due to agricultural activity upstream—there was an expansion in agriculture land and decreases in water environments over study period.
Mosquito Abundance				
Jardine et al. 2004 [20]	Kununurra, northeast of Kimberley region, Western Australia	Irrigated and non-irrigated land use; measurement unspecified	Adult and larval mosquito abundance; via biological sampling survey	Results indicate mosquito breeding associated with the irrigation area may be responsible for an increased health risk of mosquito-borne disease transmission, at the end of the dry season—a time period not hitherto a risk of mosquito-borne disease transmission.
Carver et al. 2011 [28]	Saltmarshes east and northeast of Hobart, Tasmania, Australia	Multiple; via approximation in the field, verified with aerial photographs	*Aedes camptorhynchus* (Ross River virus vector) abundance; measured via mosquito dipper samples	Mosquito abundance indirectly linked to land use via changes in abiotic and biotic predictors, for e.g., greater abundance of mosquitoes recorded in saltmarshes fringed by agricultural land (compared to natural vegetation-fringed saltmarshes) because of reduced ostracods (mosquito predators) and increases cover of samphire (positively related to mosquito abundance).
Other Infectious Disease				
Kaestli et al. 2009 [25]	Rural area within 50 km radius of Darwin, Northern Territory, Australia	Undisturbed sites and environmentally manipulated areas; via GIS	Presence of soil-dwelling saprophyte bacterium *Burkholderia pseudomallei* (as risk of melioidosis); via soil DNA extraction and targeting	While not a statistically significance difference between occurrence *of B. pseudomallei* in disturbed versus undisturbed sites, *B. pseudomallei* occurrence was significantly associated with different elements in the environment. *B. pseudomallei* was significantly associated with areas rich in grasses, whereas at environmentally disturbed sites, *B. pseudomallei* was associated with presence of livestock animals, lower soil pH (from fertilizers), and different combinations of soil texture and colour (associated with anthropogenic disturbance).
Kaestli et al. 2012 [33]	Rural Darwin, tropical Top End, Northern Territory, Australia	Native and exotic grasses; measurement unspecified	*B. pseudomallei* occurrence in plants and soil (as risk of melioidosis); via observational longitudinal field studies and laboratory-based grass inoculation experiments	*B. pseudomallei* occurs significantly more often in areas with exotic grasses (vs native)
Wellbeing				
Adams et al. 2014 [40]	Daly River catchment; Northern Territory, Australia	Proposed agricultural development in an area of multiple land use types	Perceived wellbeing; via focus groups and cross-sectional questionnaire survey	On average residents placed low importance on economic factors relative to other aspects of wellbeing (biodiversity, socio-cultural, and recreational), and were dissatisfied with proposed development and associated environmental impacts. Differences noted in preferences between Indigenous respondents and those in agriculture who would benefit more in short term from development.
Stoeckl et al. 2013 [39]	The Daly River catchment, Northern Territory, Australia	Proposed 110,400 hectares of agricultural development with additional dry season water extraction from surface water systems and groundwater projected	Wellbeing in Indigenous and non-Indigenous people; via simulated financial and socio-cultural impact	Different types of economic growth, with different levels of government sector and agricultural development, and different quantities of water used, were modelled. The high-water use agricultural development scenario has the most detrimental effect on the environment, with relatively modest financial returns. Financial returns for Indigenous people are less than those to Industry and to non-Indigenous people (up to five times as much). Indigenous people not only have more to lose from agricultural ‘development’ (which erodes natural capital) than do non-Indigenous people, but they also have significantly less to gain.
Dryland Salinity				
Mental Health				
Speldewinde et al. 2009 [26]	Rural south-western corner of Western Australia	Dryland salinity; from landscape mapping databases	Hospital admission for depression; from Western Australian Record Linkage Project	Elevated risk of hospitalisations for depression was associated with residence in areas proportionately more affected by dryland salinity. The spatial analysis also indicated the important role of socio-economic factors, and Aboriginal or Torres Strait Islander identification status as factors predictive of hospitalisation for depression.
Fearnley et al. 2014 [41]	Rural southwestern, Western Australia	Dryland salinity data; from existing landscape mapping database	Mental health; via Component Score of Short Form-36 health survey (mailed, self-reported), as part of longitudinal study	No associations found between mental health scores for women, and salinity. Area level measurements of aspects of physical environment may be poor indicators for individual health outcome analyses. At lower spatial scales, any effect of soil salinisation on human health may be difficult to determine independently of socioeconomic factors.
Mosquito Abundance				
Jardine et al. 2008 [23]	Southwestern Western Australia	Dryland salinity; via field surveys and observations	Abundance of Ross River Virus vector *Ae. Camptorhynchus*; via Encephalitis Vector Surveillance light traps baited with CO_2_.	Dryland salinity has strong influence on mosquito community structure and is significantly associated with the abundance of mosquitoes. While interaction between salinity and season is also significant, the influence of salinity remained substantial even when seasonal variation taken into account.
Carver et al. 2009 [24]	Wheat belt, Western Australian	Dryland salinity; existing categorisation, modified and supplemented by field observations	Abundance of *Ae. Camptorhynchus* (Ross River virus vector); via larvae counts from pond net samples	Increasing salinity positively related to abundance of mosquitoes. Accordingly, dryland salinity increases the zoonotic potential for Ross River virus transmission primarily by facilitating abundance of *Ae. camptorhynchus*.
Other Chronic Disease				
Speldewinde et al. 2011 [31]	Rural southwest, Western Australia	Dryland salinity; data from soil and landscape mapping database	Asthma, suicide, ischaemic heart disease, and depression; data from Western Australia’s Data Linkage Unit database	Presence of depression was consistently linked to residence in areas with high salinity and the association of asthma, suicide, and heart disease with salinity was likely attributable to the co-morbidity of the conditions with depression.
Anthropogenic Land Change—General				
Mosquito Abundance				
Claflin et al. 2017 [49]	Urban mangrove forest sites along the Parramatta River, Sydney, Australia	Multiple; via aerial photographs from Google Maps imagery	Mosquito abundance; via carbon dioxide-baited surveillance traps	The size of the mangrove stand itself had a significant positive effect on mosquito abundance—explained by resource concentration. Also positive (but not significant) relationship between percentage of industrial land and mosquito abundance. Percentage of parkland and open water had no effect on mosquito abundance and percentage of residential land and bushland in surrounding area had strong negative effect on mosquito abundance in urban mangroves—likely through changes in surface water flow in general but also due to below average rainfall preceding surveys.
Steiger et al. 2016 [47]	Wet Tropics bioregion of northeastern Australia	Multiple; via field data	Mosquito community structure; collected using Center for Disease Control and Prevention light traps	Results indicated a diverse mosquito community in tropical Australia, and community composition varies considerably between forests and disturbed habitats. Most disease transmitting species predominantly occur in grasslands created by humans, with potential implications for pathogen transmission to humans and wildlife.
Steiger et al. 2012 [34]	Tropical lowlands of north Queensland, Australia	Multiple; via field data	Mosquito community structure; collected using Center for Disease Control and Prevention light traps	Mosquito species richness was elevated in anthropogenic grasslands relative to rainforest habitats; the creation of anthropogenic grasslands adjacent to rainforests may increase the susceptibility of species in both habitats to the transmission of novel diseases via observable changes to and mixing of the vector community on rainforest edges.
Pollen Counts				
Haberle et al. 2014 [42]	11 urban centres across Australia and New Zealand	Multiple—as exist within a 100 km radius of each city’s aerobiology recording station; measurement unspecified	Pollen counts and taxa; from aerobiology recording stations across Australia and New Zealand	While urban areas from similar climate zones have similar pollen spectra but with differences due to surrounding land use and establishment of non-native plants, the only statistically significant factor explaining the difference between airborne pollen in each site, was minimum temperature and mean annual precipitation
Respiratory Disease				
Liddicoat et al. 2018 [53]	Australia-wide	Multiple; via exhaustive Australia-wide gridded mapping datasets	Respiratory disease public hospital admissions; from Social Health Atlas of Australia	Beneficial respiratory health outcomes associated with diversity of major vegetation groups, species richness, proportion of eucalypt forests proportion of open trees, diversity of land use, and proportion of nature conservation. Possible driver for this relationship is protective immunomodulatory influence from microbial diversity and other bioactive agents associated with biodiverse environments.
Vector-Borne and Infectious Disease				
Walsh et al. 2018 [54]	Australia-wide	Multiple: corresponding to geographical coordinates for the location of each Ross River virus epidemic; Google Maps Open Street Map	Epidemics of Ross River virus in humans across Australia; surveillance data	In anthropogenically impacted environments features mediating the movement of water through the landscape and the ecological niche of wildlife hosts promoted landscapes suitable to Ross River virus epidemics. Moderate soil-water balance and proximity to controlled water reservoirs were the two most influential features of Ross River virus landscape suitability.
Smith et al. 2014 [43]	Queensland and New South Wales, Australia	Normalised difference vegetation index; via meteorology data	Reported Hendra virus infection in horses; from government authorities	Variation in vegetation index did not significantly explain risk for Hendra virus infection in horses
Field et al. 2016 [45]	Boonah, 80 km southwest of Brisbane, Australia	Preferred foraging landscape-type of black flying fox; via GPS data logger	Equine exposure to Hendra virus risk; via GPS data logger of landscape utilisation of black flying-foxes and horses	Flying foxes frequently foraged in degraded remnant native vegetation and/or introduced environmental weed and garden ornamental species—consistent with increased likelihood of flying foxes around rural houses and out-buildings and directly relevant to Hendra virus spillover risk. In addition, seasonally fruiting Ficus—typically planted for stock shade and shelter, but also a favoured flying fox food resource—were present at a third of recurring foraging locations.
Walsh et al. 2017 [51]	Eastern Australia	Multiple; via moderate-resolution imaging spectroradiometer	Flying fox habitat suitability and Hendra virus spillovers to horses; from biodiversity, veterinary and government databases	Hendra virus spillovers associated with net increases in human population (human footprint) and resulting changing habitat (land cover) suitable to flying foxes
Faust et al. 2018 [52]	-	Anthropogenic land conversion; simulated (mathematical model)—not parameterised for specific locations	Pathogen spillover from wildlife to domestic animals and humans; simulated (mathematical model)—not parameterised for specific diseases	Models highlight changing host population densities and edge effects as mechanisms driving disease emergence in converted landscapes. Time since initial habitat loss, in addition to rate and scale of land conversion, drive changes in infectious disease transmission. A hump-shaped relationship of pathogen transmission between two species occurs across a gradient of land conversion, with highest disease risk at intermediate levels of habitat loss. Largest, but rarest, epidemics occur at extremes of land conversion.
Wellbeing				
Luck et al. 2011 [29]	Nine towns and cities across Victoria and New South Wales, southeastern Australia	Multiple; via field studies and satellite imagery (Advanced Land Observation Satellite) data.	Personal wellbeing, neighbourhood wellbeing, connection to nature, neighbourhood activity level, general activity level; via questionnaire survey using Wellbeing Group 2006 index and modified version of the Connectedness to Nature Scale	Measures of neighbourhood environment weakly related to residents’ personal wellbeing and level of connection to nature. Some evidence that increases in vegetation density associated with increases in personal wellbeing for certain types of residents. Conversely, many environmental variables strongly related to variation in neighbourhood wellbeing across a range of demographic categories, and residents’ satisfaction with their local neighbourhood increased with greater number of bird species, higher proportion of vegetation cover, and lower level of urban development. Nevertheless, demographic variables always the variables most strongly associated with wellbeing or connection to nature.
Urbanisation				
Risk of Zoonotic Disease				
Plowright et al. 2011 [30]	Eastern Australia	Urbanisation	Birth rate, death rate, seasonality, incidence and distribution of disease parameters, and population and meta-population characteristics of grey-headed and black flying foxes; estimated from experimental, captive and field studies	Loss to various anthropogenic land use changes of 75% of once contiguous forest cover on east coast of Australia—natural food resource of grey-headed and black flying foxes—provides plausible scenario for recent apparent increased frequency of Hendra virus outbreaks. Year-round alternative food in expanding urban and peri-urban areas increases number of flying foxes in contact with human and domestic animal populations and decreases bat migratory behaviour, which could lead to decline in bat population immunity, giving rise to more intense outbreaks after local viral reintroduction.
Contaminated Sites				
Wellbeing				
Prior et al. 2019 [56]	13 contaminated sites across urban areas in Australia	Contaminated sites; identified via relevant authorities.	Residents’ worry about disruptive effect of environmental contamination on health and well-being; via telephone questionnaire survey	Female participants, people living with disability or long-term illness, and those living close to contaminated sites were significantly more likely to worry about contamination. Presence of hydrocarbon, metal, and chlorinated solvent were significantly more likely to cause worry about contamination than asbestos. Worries were focused more on how contaminants might disrupt physical health, mental health, and lifestyle, than they were on the effect of the contaminant on flora, fauna, and broader ecosystem.
Mining				
General Health				
Werner et al. 2016 [48]	Queensland, Australia	Coal seam gas extraction, coal mining, rural/agricultural	Hospitalization rates as measured by ICD-10-AM codes; from Queensland Hospital Admitted Patient Data Collection	Coal seam gas area showed increases in hospitalization rates (compared to rural area, not to coal mining area) for neoplasms and blood/immune diseases. This descriptive-analytic study provided preliminary assessment of hospitalization rates only and did not assess causality.
McCarron 2013 [36]	Western Downs Region, Queensland, Australia	Coal seam gas mining	Self-reported or proxy self-report (for children) health; via questionnaire survey	Residents who had previously reported health concerns related to coal seam gas exposure, as well as their near neighbours, were surveyed. One third of people over 6 years reported spontaneous nose bleeds, and almost three quarters reported skin irritation. Eye irritation was reported in over half of children. One third of all children to age 18 were reported to experience paraesthesia. Almost all children aged 6-18 reported suffering from headaches and for over half of these the headaches were severe. Of people aged 6 years and over, severe fatigue and difficulty concentrating was reported for over half.
Queensland Health 2013 [38]	Western Downs Region, Queensland, Australia	Coal seam gas extraction; data from various environmental health monitoring reports—odour, fugitive emissions, and noise	Community health complaints relating to coal seam gas activity from residents; data from clinical health reports	On the basis of the clinical and environmental monitoring data included in this summary risk assessment it was concluded by authors that a clear link could not be drawn between health complaints by some residents and impacts of local coal seam gas industry on air, water or soil.
Mental Health				
Morgan et al. 2016 [46])	Australia-wide	Coal seam gas extraction	Global stress burden and mental health using adapted version of the Edinburgh Farming Stress Inventory, and Depression Anxiety Stress Scales; via questionnaire survey	For farmers recruited via agri-political organizations—New South Wales Farmers Association, Ag Force, and Lock the Gate—concern about coal seam gas impacts on human health, community, and environment (constituting ‘off-farm’), was rated, along with weather and economic viability, as most stressful factor for farmers, and explained significant amount of unique variance in farmers’ depression and stress reactivity (after controlling for more traditional agricultural stressors). Coal seam gas impacts ‘on-farm’—operations, profitability, and personal privacy—were less of a concern to farmers.
Wellbeing				
Walton et al. 2014 [44]	Western Downs Region, southern Queensland, Australia	Coal seam gas extraction	Community wellbeing, community resilience, and expected level of future wellbeing; via questionnaire survey	Overall perceptions of community wellbeing were positive notwithstanding three unsatisfactory aspects—roads, community participation in decision making, and management of environment over long term. There was dissatisfaction with planning for the future, access to relevant information, and leadership within the community (community resilience factors). Residents were not optimistic about the future and expected decline in wellbeing.
Albrecht et al. 2007 [21]	Upper Hunter region of New South Wales, Australia	Open-cut coal mining and power industries	Perceived threats to wellbeing and actual lived experience; via key informant, community member, and focus group interviews	Transformation of the regional landscape challenged many participants’ sense of place, identity, physical and mental health, and general wellbeing. Participants also felt powerless to influence the change process. These responses resonate with the dominant components of ‘solastalgia’, which was experienced even by some who worked in the relevant mining and power industries.
Moffatt et al. 2013 [37]	Clarence–Moreton coal basin, Queensland, Australia	Mining development proposal: large open-cut coal mine	Perceived psychological impacts for individuals and community; via semi-structured interviews	Semi-structured interviews with range of individuals representing various roles and service industries within the community indicated that the proposed mineral development was source of psychological stress, in farmers in particular. Impacts at individual and family level included a sense of powerlessness; unknown future; interrupted succession plans for young and old; threat of disruption to relationship with the land and landscape; and changed financial circumstances. The proposal also created tensions within the community regarding differential impacts, with some seeing opportunity and others perceiving threat.
Gillespie et al. 2012 [32]	Hunter Valley Coalfields, New South Wales, Australia	Proposed extension of open-cut coal mine	Wellbeing; via choice modelling questionnaire	Community well-being declined with increased clearing of endangered ecological communities, loss of highly significant Aboriginal sites, and displacement of rural families from affected villages, but increased with length of time that mine provides employment and with planting or protection of endangered ecological communities as offsets.
Traditionally-Owned Ancestral Lands				
General Health and Wellbeing				
Johnston et al. 2007 [22]	Arnhem land, northeast corner of Northern Territory, Australia	Traditionally owned Indigenous ancestral lands	Perspectives of health and wellbeing; via observation and unstructured and semi-structured interviews	Major themes which emerged from the observations and interviews with Aboriginal community members, included: the need that both land and people have for each other, for the well-being of both; that traditional lands provided access to traditional food and medicines (and physical activity to get them); a way of escaping from stresses; a means to educating young people; and deeper connection to the past and therefore to Aboriginal identity via traditional stories and beliefs.
‘Yotti’ Kingsley et al. 2009 [27]	Victoria, Australia	Traditional lands of three Australian Aboriginal language groups	Health and wellbeing; via semi-structured interviews	Semi-structured interviews identified that Caring for Country builds self-esteem, fosters self-identity, maintains cultural connection, and enables relaxation and enjoyment through contact with the natural environment. Caring for Country—a traditional practice of over 50,000 years—constitutes a key determinant of the health and wellbeing of Indigenous people in Australia.

## References

[1] Myers S.S., Gaffikin L., Golden C.D., Ostfeld R.S., Redford K.H., Ricketts T.H., Turner W.R., Osofsky S.A. (2013). Human health impacts of ecosystem alteration. Proc. Natl. Acad. Sci. USA.

[2] Whitmee S., Haines A., Beyrer C., Boltz F., Capon A.G., Dias B.F.D.S., Ezeh A., Frumkin H., Gong P., Head P. (2015). Safeguarding human health in the Anthropocene epoch: Report of The Rockefeller Foundation–Lancet Commission on planetary health. Lancet.

[3] Intergovernmental Panel on Climate Change (2019). Summary for Policymakers. Climate Change and Land: IPCC Special Report on Climate Change, Desertification, Land Degradation, Sustainable Land Management, Food Security, and Greenhouse Gas Fluxes in Terrestrial Ecosystems.

[4] IPBES (2019). Summary for Policymakers of the Global Assessment Report on Biodiversity and Ecosystem Services of the Intergovernmental Science-Policy Platform on Biodiversity and Ecosystem Services.

[5] Australian Goverment Department of Agriculture (2019). Land Use Mapping: Commonwealth of Australia. https://www.agriculture.gov.au/abares/aclump/land-use/land-use-mapping.

[6] Lesslie R., Thackway R., Smith J. (2010). A National-Level Vegetation Assets, States and Transitions (VAST) Dataset for Australia (version 2.0).

[7] Metcalfe D.J., Bui E.N. (2017). Australia State of the Environment 2016: Land, Independent Report to the Australian Government Minister for the Environment and Energy.

[8] Bryan B.A., Nolan M., McKellar L., Connor J.D., Newth D., Harwood T., King D., Navarro J., Cai Y., Gao L. (2016). Land-use and sustainability under intersecting global change and domestic policy scenarios: Trajectories for Australia to 2050. Glob. Environ. Chang..

[9] Bradshaw C.J.A. (2012). Little left to lose: Deforestation and forest degradation in Australia since European colonization. J. Plant Ecol..

[10] McFarlane R.A., Sleigh A.C., McMichael A.J. (2013). Land-use change and emerging infectious disease on an island continent. Int. J. Environ. Res. Public Health.

[11] Kessler M.K., Becker D.J., Peel A.J., Justice N.V., Lunn T., Crowley D.E., Jones D.N., Eby P., Sánchez C.A., Plowright R.K. (2018). Changing resource landscapes and spillover of Henipa viruses. Ann. N. Y. Acad. Sci..

[12] Páez D.J., Restif O., Eby P., Plowright R.K. (2018). Optimal foraging in seasonal environments: Implications for residency of Australian flying foxes in food-subsidized urban landscapes. Philos. Trans. R. Soc. B Biol. Sci..

[13] Levac D., Colquhoun H., Nixon S.A., Levac D., Colquhoun H., Nixon S.A. (2010). Scoping studies: Advancing the methodology. Implement. Sci..

[14] Mastel M., Bussalleu A., Paz-Soldan V.A., Salmón-Mulanovich G., Valdés-Velásquez A., Hartinger-Peña S.M. (2018). Critical linkages between land use change and human health in the Amazon region: A scoping review. PLoS ONE.

[15] Arksey H., O’Malley L. (2005). Scoping studies: Towards a methodological framework. Int. J. Soc. Res. Methodol..

[16] Coutts C., Hahn M. (2015). Green infrastructure, ecosystem services, and human health. Int. J. Environ. Res. Public Health.

[17] Corvalan C., Hales S., McMichael A.J. (2005). Ecosystems and human wellbeing: Health synthesis. Millennium Ecosystem Assessment.

[18] Australian Government Department of Health Australian National Notifiable Diseases by Disease Type 2019. https://www1.health.gov.au/internet/main/publishing.nsf/Content/cda-surveil-nndss-casedefs-distype.htm.

[19] Watson R.T., Noble I.R., Bolin B., Ravindranath N.H., Verardo D.J., Dokken D.J. (2000). Land Use, Land-Use Change and Forestry: A Special Report of the Intergovernmental Panel on Climate Change.

[20] Jardine A., Lindsay M., Heyworth J., Weinstein P. (2004). Dry-season mosquito breeding associated with irrigation in the Northeast Kimberley Region of Western Australia: Potential impact on mosquito-borne disease transmission. EcoHealth.

[21] Albrecht G., Sartore G.-M., Connor L., Higginbotham N., Freeman S., Kelly B., Stain H., Tonna A., Pollard G. (2007). Solastalgia: The distress caused by environmental change. Australas. Psychiatry.

[22] Johnston F.H., Jacups S.P., Vickery A.J., Bowman D.M.J.S. (2007). Ecohealth and aboriginal testimony of the nexus between human health and place. EcoHealth.

[23] Jardine A., Lindsay M.D., Johansen C.A., Cook A., Weinstein P. (2008). Impact of dryland salinity on population dynamics of vector mosquitoes (Diptera: Culicidae) of Ross River virus in inland areas of southwestern Western Australia. J. Med. Entomol..

[24] Carver S., Spafford H., Storey A., Weinstein P. (2009). Dryland salinity and the ecology of Ross River virus: The ecological underpinnings of the potential for transmission. Vector-Borne Zoonotic Dis..

[25] Kaestli M., Mayo M., Harrington G., Ward L., Watt F., Hill J.V., Cheng A.C., Currie B.J. (2009). Landscape changes influence the occurrence of the melioidosis bacterium Burkholderia pseudomallei in soil in Northern Australia. PLoS Negl. Trop. Dis..

[26] Speldewinde P., Cook A., Davies P., Weinstein P. (2009). A relationship between environmental degradation and mental health in rural Western Australia. Health Place.

[27] Kingsley J.Y., Townsend M., Phillips R., Aldous D. (2009). “If the land is healthy … it makes the people healthy”: The relationship between caring for Country and health for the Yorta Yorta Nation, Boonwurrung and Bangerang Tribes. Health Place.

[28] Carver S., Goater S., Allen G.R., Rowbottom R.M., Fearnley E., Weinstein P. (2011). Relationships of the Ross River virus (Togoviridae: Alphavirus) vector, Aedes camptorhynchus (Thomson) (Diptera: Culicidae), to biotic and abiotic factors in saltmarshes of south-eastern Tasmania, Australia: A preliminary study. Aust. J. Èntomol..

[29] Luck G.W., Davidson P., Boxall D., Smallbone L. (2011). Relations between urban bird and plant communities and human well-being and connection to nature. Conserv. Biol..

[30] Plowright R.K., Foley P., Field H.E., Dobson A.P., Foley J.E., Eby P., Daszak P. (2011). Urban habituation, ecological connectivity and epidemic dampening: The emergence of Hendra virus from flying foxes (*Pteropus* spp.). Proc. R. Soc. B Boil. Sci..

[31] Speldewinde P., Cook A., Davies P., Weinstein P. (2011). The hidden health burden of environmental degradation: Disease comorbidities and dryland salinity. EcoHealth.

[32] Gillespie R., Bennett J. (2012). Valuing the environmental, cultural and social impacts of open-cut coal mining in the Hunter Valley of New South Wales, Australia. J. Environ. Econ. Policy.

[33] Kaestli M., Schmid M., Mayo M., Rothballer M., Harrington G., Richardson L., Hill A., Hill J., Tuanyok A., Keim P. (2012). Out of the ground: Aerial and exotic habitats of the melioidosis bacterium Burkholderia pseudomallei in grasses in Australia. Environ. Microbiol..

[34] Steiger D.M., Johnson P., Hilbert D.W., Ritchie S., Jones D., Laurance S.G. (2012). Effects of landscape disturbance on mosquito community composition in tropical Australia. J. Vector Ecol..

[35] Baral H., Keenan R.J., Fox J.C., Stork N.E., Kasel S. (2013). Spatial assessment of ecosystem goods and services in complex production landscapes: A case study from south-eastern Australia. Ecol. Complex..

[36] McCarron G. Symptomatology of a Gas Field: An Independent Health Survey in the Tara Rural Residential Estates and Environs. https://www.gabpg.org.au/wp-content/uploads/2013/11/2013-04-symptomatology_of_a_gas_field_Geralyn_McCarron.pdf.

[37] Moffatt J., Baker P. (2013). Farmers, mining and mental health: The impact on a farming community when a mine is proposed. Rural Soc..

[38] Queensland Health Coal Seam Gas in the Tara Region: Summary Risk Assessment of Health Complaints and Environmental Monitoring Data. https://www.parliament.qld.gov.au/documents/tableoffice/tabledpapers/2013/5413t2306.pdf.

[39] Stoeckl N., Jackson S., Pantus F., Finn M., Kennard M.J., Pusey B.J. (2013). An integrated assessment of financial, hydrological, ecological and social impacts of ‘development’ on Indigenous and non-indigenous people in northern Australia. Biol. Conserv..

[40] Adams V.M., Pressey R.L., Stoeckl N. (2014). Navigating trade-offs in land-use planning: Integrating human well-being into objective setting. Ecol. Soc..

[41] Fearnley E.J., Magalhães R.J.S., Speldewinde P., Weinstein P., Dobson A. (2014). Environmental correlates of mental health measures for women in western Australia. EcoHealth.

[42] Haberle S.G., Bowman D.M.J.S., Newnham R.M., Johnston F.H., Beggs P.J., Buters J., Campbell B., Erbas B., Godwin I., Green B.J. (2014). The macroecology of airborne pollen in Australian and New Zealand urban areas. PLoS ONE.

[43] Smith C., Skelly C., Kung N., Roberts B., Field H. (2014). Flying-fox species density-A spatial risk factor for Hendra virus infection in horses in Eastern Australia. PLoS ONE.

[44] Walton A., McCrea R., Leonard R. (2014). CSIRO Survey of Community Wellbeing and Responding to Change: CSIRO: Western Downs Region in Queensland, Australia. https://www.researchgate.net/profile/Rod_Mccrea/publication/312043695_CSIRO_survey_of_Community_Wellbeing_and_responding_to_change_Western_Downs_region_in_Queensland/links/586c769008ae8fce4919e776.pdf.

[45] Field H.E., Smith C.S., De Jong C.E., Melville D., Broos A., Kung N., Thompson J., Dechmann D.K.N. (2016). Landscape utilisation, animal behaviour and Hendra virus risk. EcoHealth.

[46] Morgan M.I., Hine D.W., Bhullar N., Dunstan D.A., Bartik W. (2016). Fracked: Coal seam gas extraction and farmers’ mental health. J. Environ. Psychol..

[47] Steiger D.B.M., Ritchie S.A., Laurance S.G.W. (2016). Mosquito communities and disease risk influenced by land use change and seasonality in the Australian tropics. Parasites Vectors.

[48] Werner A.K., Watt K., Cameron C.M., Vink S., Page A., Jagals P. (2015). All-age hospitalization rates in coal seam gas areas in Queensland, Australia, 1995–2011. BMC Public Health.

[49] Claflin S.B., Webb C.E. (2017). Surrounding land use significantly influences adult mosquito abundance and species richness in urban mangroves. Wetl. Ecol. Manag..

[50] Vandandorj S., Eldridge D.J., Travers S.K., Delgado-Baquerizo M. (2017). Contrasting effects of aridity and grazing intensity on multiple ecosystem functions and services in Australian woodlands. Land Degrad Dev..

[51] Walsh M.G., Wiethoelter A., Haseeb M.A. (2017). The impact of human population pressure on flying fox niches and the potential consequences for Hendra virus spillover. Sci. Rep..

[52] Faust C.L., McCallum H.I., Bloomfield L.S.P., Gottdenker N.L., Gillespie T.R., Torney C.J., Dobson A.P., Plowright R.K. (2018). Pathogen spillover during land conversion. Ecol. Lett..

[53] Liddicoat C., Bi P., Waycott M., Glover J., Lowe A.J., Weinstein P. (2018). Landscape biodiversity correlates with respiratory health in Australia. J. Environ. Manag..

[54] Walsh M.G., Webb C. (2018). Hydrological features and the ecological niches of mammalian hosts delineate elevated risk for Ross River virus epidemics in anthropogenic landscapes in Australia. Parasites Vectors.

[55] Wijesiri B., Deilami K., Goonetilleke A. (2018). Evaluating the relationship between temporal changes in land use and resulting water quality. Environ. Pollut..

[56] Prior J.H., Gorman-Murray A., McIntyre E., Connon I., Adams J., Madden B. (2019). A geography of residents’ worry about the disruptive effects of contaminated sites. Geogr. Res..

[57] Plowright R.K., Parrish C.R., McCallum H., Hudson P.J., Ko A.I., Graham A.L., Lloyd-Smith J.O. (2017). Pathways to zoonotic spillover. Nat. Rev. Genet..

[58] Haswell-Elkins M., Bethmont A. (2016). Health concerns associated with unconventional gas mining in rural Australia. Rural. Remote. Health..

[59] Maher P. (1999). A review of ’traditional’ aboriginal health beliefs. Aust. J. Rural. Health.

[60] Australian Government Attorney-General’s Department (2019). Native Title: Australian Government. https://www.ag.gov.au/nativetitle.

[61] Garnett S., Sithole B., Whitehead P.J., Burgess C.P., Johnston F.H., Lea T. (2009). Healthy country, healthy people: Policy implications of links between indigenous human health and environmental condition in tropical Australia. Aust. J. Public Adm..

[62] Lutschini M. (2005). Engaging with holism in Australian Aboriginal health policy-A review. Aust. N. Z. Health Policy.

[63] Adame F., Arthington A.H., Waltham N.J., Hasan S., Selles A., Ronan M. (2019). Managing threats and restoring wetlands within catchments of the Great Barrier Reef, Australia. Aquat. Conserv. Mar. Freshw. Ecosyst..

[64] Gell P., Reid M., Wilby R.L. (2019). Management pathways for the floodplain wetlands of the southern Murray-Darling basin: Lessons from history. River Res. Appl..

[65] Bryan B.A., Kandulu J.M. Cost-Effective Alternatives for Mitigating Cryptosporidium Risk in Drinking Water and Enhancing Ecosystem Services. https://agupubs.onlinelibrary.wiley.com/doi/full/10.1029/2008WR007606.

[66] Hooper J., Marx S. (2018). A global doubling of dust emissions during the Anthropocene?. Glob. Planet. Chang..

[67] Marx S.K., Kamber B.S., McGowan H., Denholm J. (2011). Holocene dust deposition rates in Australia’s Murray-Darling Basin record the interplay between aridity and the position of the mid-latitude westerlies. Quat. Sci. Rev..

[68] Marx S.K., McGowani H., Kamber B.S., Knight J.M., Denholm J., Zawadzki A. (2014). Unprecedented wind erosion and perturbation of surface geochemistry marks the Anthropocene in Australia. J. Geophys. Res. Earth Surf..

